# Advances of Organosulfur Materials for Rechargeable Metal Batteries

**DOI:** 10.1002/advs.202103989

**Published:** 2021-11-25

**Authors:** Wei Guo, Dan‐Yang Wang, Qiliang Chen, Yongzhu Fu

**Affiliations:** ^1^ College of Chemistry Zhengzhou University Zhengzhou 450001 P. R. China

**Keywords:** cathode, electrolyte additive, hybrid materials, organosulfur, rechargeable lithium batteries

## Abstract

Battery materials have become a hotspot in the academic research. Organosulfur compounds are considered as a promising class of cathode materials for rechargeable metal batteries. They have attracted increasing attention in recent years after a long‐term stagnancy since 1980s. Recent studies have focused on the understanding of redox mechanism of linear organosulfur molecules R‐S*_n_*‐R with defined structures. In addition, some new organosulfur compounds are developed. The reversible sulfur—sulfur (S—S) bond breakage/formation of organosulfur in batteries makes them applicable as functional materials in batteries. In this review, new organosulfur materials including molecules, polymers, and composites are introduced. In the following, organosulfur‐inorganic hybrid materials are discussed, which have shown unique redox process and enhanced battery performance. In the third part, organosulfur additives are used in Li‐S batteries, which can improve the formation of solid‐electrolyte interphase (SEI) and alter the redox pathways of sulfur cathodes. In the fourth part, organosulfur materials used in other metal batteries are introduced. Lastly, a summary and some perspectives are given. This review presents an overview of the recent advances of organosulfur materials in batteries and provides guidance for the future development of these materials.

## Graphical abstract

In this review, recent understanding and developments of organosulfur for rechargeable lithium batteries are summarized, including molecules, polymers, composites, and organosulfur‐inorganic hybrid materials. In addition, organosulfur as electrolyte additives in Li‐S battery and other metal batteries are covered. Finally, some perspectives are given. This review provides guidance for the future development of organosulfur materials for batteries.

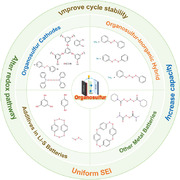

## 1 Introduction

Lithium‐ion (Li‐ion) batteries have dominated the power supply market of portable electronics and electric vehicles because of their high specific energy (Wh kg^−1^) and energy densities (Wh L^−1^). The current cathode materials are mainly crystal materials such as layered LiCoO_2_, spinel LiMn_2_O_4_, and olivine LiFePO_4_.^[^ ^1^ ^]^ Their structures allow reversible lithium ion intercalation/deintercalation, offering long cycle life for practical application. However, the theoretical specific capacities and energies of these materials are limited to below 250 mAh g^−1^ and 800 Wh kg^−1^, respectively. To further increase these values, alternative electrode materials with higher specific capacities are needed. Significant efforts are being focused on transition metal oxides, e.g., high‐nickel‐layered oxide cathodes.^[^ ^2^ ^]^ In recent years, rechargeable lithium metal batteries have attracted tremendous interests because of the high capacity of lithium metal anode (3862 mAh g^−1^). To couple with lithium metal anode, lithium‐free cathode materials such as sulfur,^[^ ^3^ ^]^ oxygen,^[^ ^4^ ^]^ and organic compounds^[^ ^5^ ^]^ can be used. Although there are many challenges associated with the lithium metal anode, such as dendrite formation and safety concern,^[^ ^6^ ^]^ these rechargeable lithium metal batteries are definitely promising to overcome the energy limits of Li‐ion batteries. A lot of efforts are being focused on solving the issues of lithium metal anodes.^[^ ^7^ ^]^ At the same time, alternative lithium‐free cathode materials are also attracting persistent attention.

**Figure 1:**
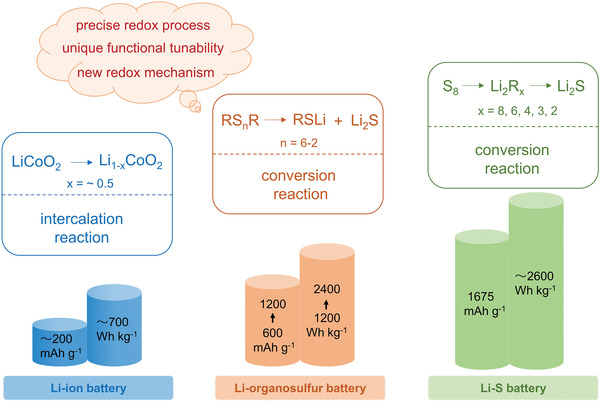
Specific capacity and specific energy comparison of Li‐ion, Li‐organosulfur, and Li‐S batteries.

Among the various cathode materials, elemental sulfur is one of the most promising candidates because of the high capacity of 1675 mAh g^−1^ leading to the high specific energy of 2600 Wh kg^−1^ of lithium‐sulfur (Li‐S) batteries. In a Li‐S battery, many challenges exist facing the lithium metal anode as well as the sulfur cathode. There have been several thorough and in‐depth reviews about the issues and possible solutions for Li‐S batteries.^[^ ^8^ ^]^ As a class of sulfur derivatives, organosulfur as cathode materials has attracted much attention.^[^ ^9^ ^]^ **Figure** **1** shows the specific capacity and energy comparison of Li‐ion, Li‐organosulfur, and Li‐S batteries. The capacity and specific energy of Li‐organosulfur batteries are competitive compared with those of Li‐ion batteries. They have additional benefits such as precise redox processes, new redox mechanism, and unique functional tunability, compared with Li‐S batteries. Since the first study by Visco and DeJonghe in 1980s,^[^ ^10^ ^]^ not much progress has been made. In recent years, growing interest in organosulfur materials for rechargeable metal batteries has emerged.^[^ ^11^ ^]^ The linear organosulfur compounds R‐S*_n_*‐R (*n* = 2–8) have become models for understanding the redox mechanism because of their defined structure.^[^ ^9c^ ^]^ The R group have profound effects on the electrochemical and physical properties of organosulfur molecules.^[^ ^11^, ^12^ ^]^ The studies on organosulfur materials not only expand the sulfur cathode family, but also improve our understanding of electrochemistry. The gains from these studies also help develop advanced materials for practical applications.

In this review, we summarize the recent advances of organosulfur materials in rechargeable lithium batteries mostly after year 2019. In the first section, the newly developed organosulfur molecules and polymers are presented. They include 1,4‐bis(diphenylphosphanyl) tetrasulfide synthesized by an electrochemical method and hyperbranced polymer based on the condensation reaction between 1,3,5‐benzentrithiol and elemental sulfur. Two organosulfide–metal complexes are introduced. To enhance the conductivity and improve the cycling stability of organosulfur electrodes, some strategies to incorporate carbon materials are discussed. In the second section, several organosulfur–inorganic hybrid cathodes are described in detail, in which organosulfur plays an important role and interesting redox processes are revealed. In the third section, organosulfur materials used as additives in Li‐S batteries are described. In the last section, organosulfur materials used in other battery systems are briefly introduced, such as redox flow batteries and sodium (Na)/magnesium (Mg) batteries. Finally, a summary and some perspectives about the future development of organosulfur materials are given.

## 2 Organosulfur Cathodes

Organosulfur consists of organic groups R which are bonded with S—S bonds. Sulfur could be bonded with the R group via di, tri, or even multiple sites, making organosulfur materials highly diversified. They can be small molecules, linear, or hyperbranched polymers. Between R groups, there could be single S—S bonds or a sulfur chain ‐S*_n_*‐, where *n* > 2. The specific capacity of organosulfur lies in the number of S—S bonds and sulfur contents in the structure. Our previous paper provides a comprehensive review on organosulfur (organosulfides) as cathode materials for rechargeable lithium batteries in 2019.^[^ ^9c^ ^]^ Some small molecules such as dimethyl and diphenyl polysulfides, thiuram polysulfides, and selenium‐containing organosulfides have been covered. In this section, we only focus on organosulfur materials recently developed. In addition, some organosulfide–metal complexes show unique physical and electrochemical properties. Moreover, the efforts to enhance the conductivity of organosulfur electrodes by introducing different carbon materials are covered as well.

### 2.1 Organosulfur Molecules

There are few organosulfur molecules that have been studied in rechargeable lithium batteries since 2019. First of all, it is quite challenging to synthesize pure or even analytical pure organosulfur molecules, in particular organosulfur molecules with long sulfur chains (*n* > 2). In addition, some of them are not stable in air, posing challenges for handling these chemicals. In addition, the electrochemical conversion processes of organosulfur molecules in rechargeable batteries are usually quite complex, making the characterization difficult. Below we list some organosulfur molecules investigated in rechargeable batteries recently.

**Figure 2:**
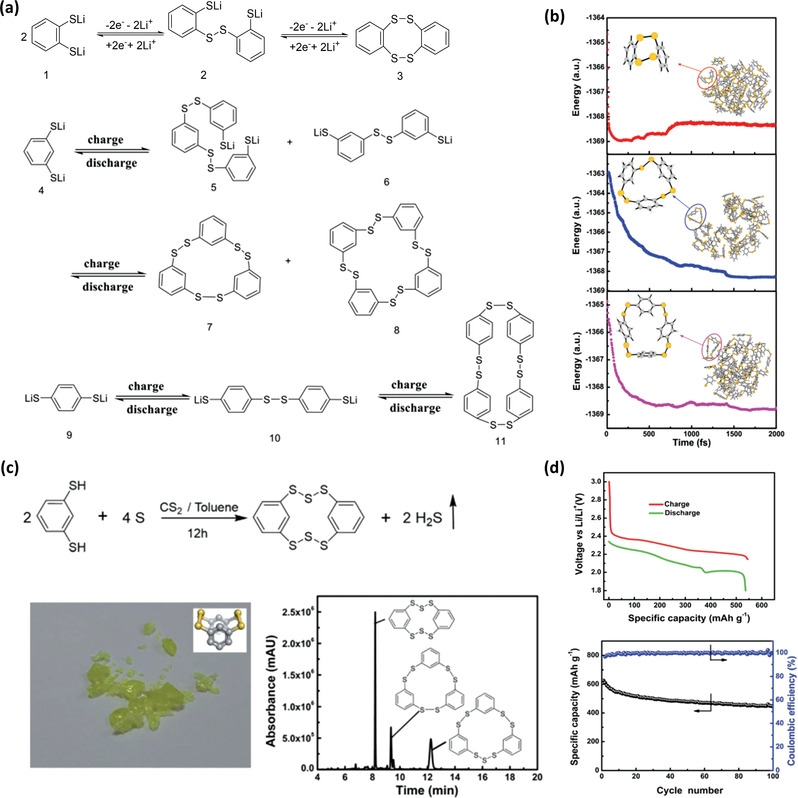
a) Redox reactions of 1,2‐LBDT, 1,3‐LBDT, and 1,4‐LBDT in lithium batteries. b) The trajectories, obtained with the GFN‐xTB simulation of 1,2‐LBDT, 1,3‐ LBDT, and 1,4‐ LBDT starting from 64 equilibrium molecules for each, showing a downhill pathway. Reproduced with permission.^[^ ^13^ ^]^ Copyright 2019, Wiley‐VCH. c) Synthesis of HDBCO, optical image of the synthesized material, UV absorption spectrum of the sample in UPLC‐QTof‐MS. d) Voltage profile of a Li/HDBCO cell at C/2 rate and cycling performance of the cell at C/10 rate. Reproduced with permission.^[^ ^14^ ^]^ Copyright 2020, Royal Society of Chemistry.

Li et al. investigated three lithium benzenedithiolate (LBDT) isomers, i.e., 1,2‐LBDT, 1,3‐LBDT, and 1,4‐LBDT.^[^ ^13^ ^]^ The sulfur bonding sites have profound effects on the recharged products and their electrochemical behavior in lithium batteries. 1,2‐LBDTs are mostly converted to dimers in the charge, 1,3‐LBDTs are converted to trimers and tetramers, and 1,4‐LBDT are primarily converted to tetramers because of the different stability energy of the charged products, as shown in **Figure** **2**a. Simulation also confirms the feasibility of forming these cyclic compounds between benzenedithiolate radicals (Figure 2b). During the discharge process, two distinguishable discharge voltage plateaus can be observed. 1,2‐LBDT offers faster reaction kinetics than the other two molecules, in terms of high‐rate capability and high mass loading performance. In the following work, Li et al. utilized the condensation reactions between 1,3‐benzenedithiol and elemental sulfur to synthesize an intermolecular cyclic organosulfur compound with multiple S—S bonds, i.e., 2,3,4,6,7,8‐hexathia‐1,5(1,3)‐dibenzenacyclooctaphane (HDBCO), as shown in Figure 2c.^[^ ^14^ ^]^ Each HDBCO molecule can take 8 Li^+^ and e^−^, offering a high theoretical specific capacity of 622.3 mAh g^−1^. The synthesized products include two other cyclic organosulfur compounds, as shown in the MS spectrum in Figure 2c. The synthesized material shows multiple discharge voltage plateaus and reasonable cycling performance (Figure 2d). As the battery cycles, the recharged products vary in terms of their contents in the electrodes.

**Figure 3:**
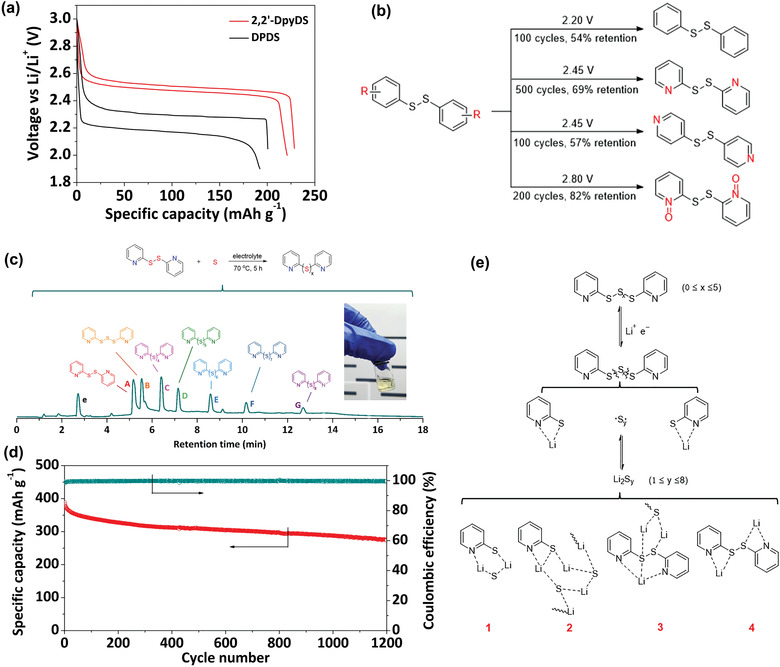
a) Voltage profile of Li/2,2′‐DpyDS and Li/DPDS cells at C/10 rate. b) Electrochemical behavior comparison of DPDS, 2,2′‐DpyDS, and 4,4′‐DpyDS. Reproduced with permission.^[^ ^16^ ^]^ Copyright 2019, Royal Society of Chemistry. c) The synthesis of a spectrum of dipyridyl polysulfides in electrolyte and TIC spectrum of the mixture catholyte. d) Long‐term cycling performance of a Li/Py_2_S*_x_* cell at 1 C rate. e) Illustration of the proposed lithiation process in lithium battery, complexes 1–4 are either simulated or detected by UPLC‐QTof‐MS. Reproduced with permission.^[^ ^18^ ^]^ Copyright 2020, Wiley‐VCH.

Organosulfur molecules containing N‐heterocycles such as thiuram polysulfides have shown unique electrochemical behavior in lithium batteries.^[^ ^15^ ^]^ The lone pair of electrons on N atoms has strong electrostatic interaction with lithium ions, changing the behavior of discharged products in batteries. To reveal the effect of N‐heterocycles, dipyridyl disulfide (DpyDS) was investigated in comparison with diphenyl disulfide (DPDS) by Wang et al.^[^ ^16^ ^]^ With only two carbon replaced by N atoms, DpyDS shows significantly improved performance in terms of discharge voltage (2.45 V vs 2.2 V of DPDS, **Figure** **3**a) and cycling stability (69% capacity retention after 500 cycles vs 54% capacity retention of 100 cycles of DPDS, Figure 3b). Interestingly, only 2,2′‐DpyDS shows such enormous performance difference, whereas 4,4′‐DpyDS does not. Density functional theory (DFT) calculations reveal that the discharged product of 2,2‐DpyDS forms a tight cluster‐like network reducing its dissolution and loss in the liquid electrolyte. Another study done by Fan et al. shows that 2,2′‐DPyDS in the ether electrolyte is inert in the chemical reaction with Li^0^, whereas DPDS is not.^[^ ^17^ ^]^ When in contact with lithiated graphite carbon paper (Li‐CP) for 10 days, only 6.3% of DPyDS is reduced by Li‐CP, but 58.7% of DPDS is reduced. This comparison reveals another reason behind the ultrastable cycling performance of DPyDS in rechargeable lithium metal batteries. Therefore, it can serve as an active material in the catholyte of a membrane‐free redox flow battery, leading to stable cycling performance.

Wang et al. further synthesized dipyridyl polysulfide by the addition reaction between DpyDS and elemental sulfur, as shown in Figure 3c.^[^ ^18^ ^]^ The target molecule is dipyridyl trisulfide Py_2_S_3_, however, a series of dipyridyl polysulfides including tri, tetra, penta, hexa, hepta, and octasulfides are detected in the synthesized catholyte. The Li cell shows a long discharge voltage plateau at 2.45 V followed by a sulfur‐like discharge voltage profile. After recharge, similar dipyridyl polysulfides are formed based on the high‐performance liquid chromatography‐quadrupole‐time of flight mass spectroscopy (HPLC‐QTof‐MS) analysis. The cell shows a superlong cycle life of 1200 cycles with >70% capacity retention at 1 C rate (Figure 3d). The outstanding performance is related to the electrostatic interactions between N atoms of pyridyl groups and lithium ions nearby, as shown in Figure 3e. The complexes formed enable reduced loss of active materials upon cycling, leading to long cycle life.

**Figure 4:**
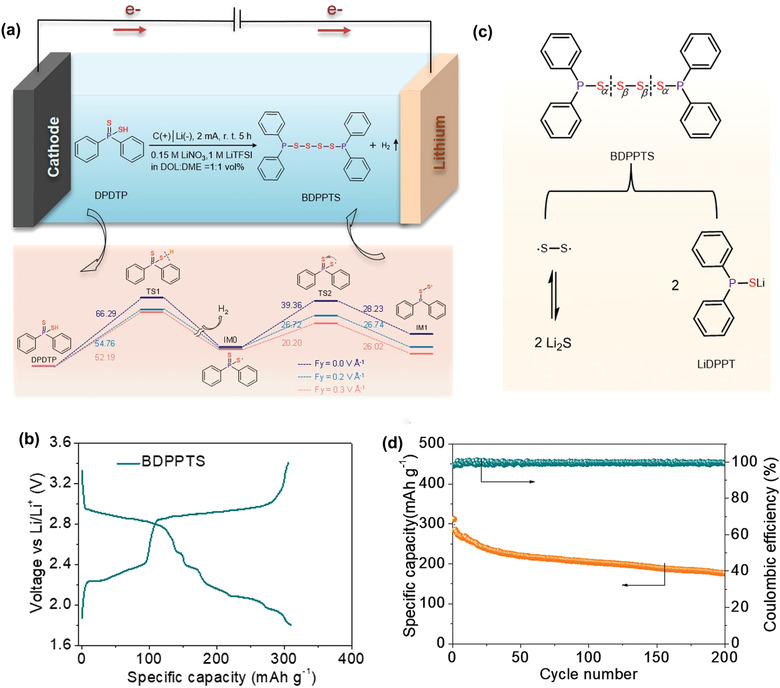
a) Synthesis of BDPPTS by electrochemical oxidation of diphenyl dithiophosphinic acid and the proposed reaction process under external electric field. b) Voltage profile of a Li/BDPPTS cell at C/10 rate. c) Proposed electrochemical redox mechanism of BDPPTS in the Li half‐cell. d) Cycling performance of a Li/BDPPTS cell at C/5 rate. Reproduced with permission.^[^ ^19^ ^]^ Copyright 2021, Nature Publishing Group.

In order to expand the organosulfur family, new compounds need to be synthesized. Wang et al. synthesized a new organosulfur molecule named 1,4‐bis(diphenylphosphanyl) tetrasulfide (BDPPTS) via an electrochemical synthesis method, as shown in **Figure** **4**a.^[^ ^19^ ^]^ Diphenyl dithiophosphinic acid was used as the precursor. The thiol group is dehydrogenated under an electric field, then the sulfur radical is formed, which is added to the P═S bond nearby forming the tricoordinate intermediate (IM1). Two IM1s are dimerized to form BDPPTS. The obtained liquid product was characterized and confirmed by Fourier transform infrared (FTIR), Raman spectroscopy, NMR, and MS analysis. The synthesized BDPPTS was evaluated in a Li cell, which shows a high discharge voltage plateau at ≈2.9 V followed by sulfur‐like voltage profile (Figure 4b). It is because the strong electron withdrawing effect of the two phenyl groups bonded with a P atom. The initial discharge capacity is 309 mAh g^−1^, which accounts for 95.7% of the theoretical capacity of BDPPTs. Based on the analysis of the discharged product, the discharge process starts with the cleavage of the S_*α*_—S_*β*_ bond followed by the S_*β*_—S_*β*_ bond (Figure 4c). The middle two sulfur radicals behave like elemental sulfur in the discharge. The battery can be cycled 200 times with 57% capacity retention (Figure 4d). When the discharge cutoff voltage is increased to 2.5 V, the cell shows a stable performance with 74.8% capacity retention after 500 cycles.

**Figure 5:**
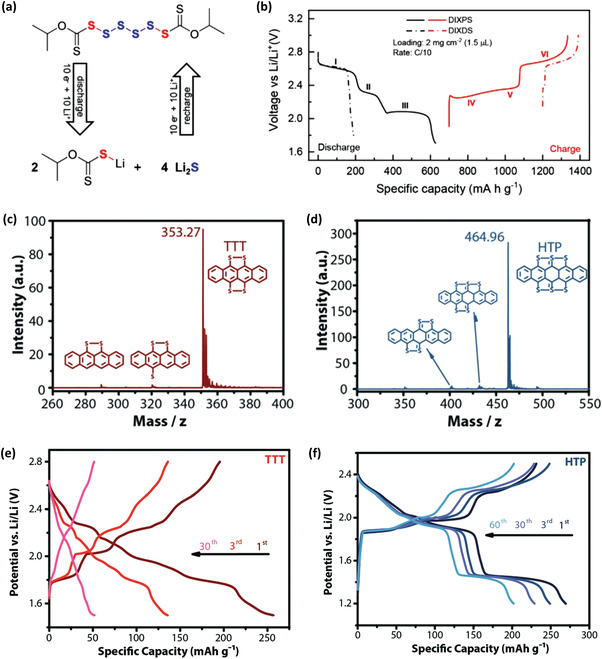
a) The redox reactions of DIXPS occurring in lithium battery. b) Voltage profile of Li/DIXPS and Li/DIXDS cells at C/10 rate. Reproduced with permission.^[^ ^20^ ^]^ Copyright 2020, Wiley‐VCH. MALDI‐TOF mass spectra of c) TTT and d) HTP. d) Voltage profile of Li/TTT and Li/HTP cells. Reproduced with permission.^[^ ^21^ ^]^ Copyright 2019, Wiley‐VCH.

Another two organosulfur model compounds are diisopropyl xanthogen disulfide (DIXDS) and diisopropyl xanthogen polysulfide (DIXPS) studied by Bhargav and Manthiram.^[^ ^20^ ^]^ Each DIXPS molecule can take 10 Li^+^ and e^−^ as shown in **Figure** **5**a, offering a high theoretical specific capacity of 672 mAh g^−1^ and specific energy of 1546 Wh kg^−1^. In a Li cell, it exhibits three discharge voltage plateaus at 2.61, 2.30, and 2.08 V with the initial discharge specific capacity of 628 mAh g^−1^ (93.5% of the theoretical capacity) at C/10 rate (Figure 5b), offering a high energy density of 1313 Wh kg^−1^ and 1694 Wh L^−1^. The cell shows a prolonged cycling (1000 cycles) stability at 4 C rate. In a pouch cell, it shows quite stable performance when tested under high‐loading (e.g., 13 mg cm^−2^) and lean‐electrolyte (e.g., electrolyte/DIXPS ratio = 2 µL mg^−1^) conditions. In addition to the outstanding performance, DIXPS is proved to be a promising sustainable material for high‐energy lithium batteries, which is in sharp contrast to the materials currently used in Li‐ion batteries, e.g., Co and Ni with potential supply shortage.

Recently, two organosulfur acenes, i.e., tetrathiotetracene (TTT) and hexathiapentacene (HTP) as shown in Figure 5c,d, have been synthesized and studied in lithium batteries by Hu et al.^[^ ^21^ ^]^ Single crystals of TTT and HTP were synthesized in a zone‐melting chemical‐vapor‐transport apparatus by a solvent‐free and continuous synthetic protocol. They show quite different discharge/charge behavior (Figure 5e,f) and cycling stability, HTP is better than TTT (72% capacity retention after 100 cycles vs 19.8% capacity retention after 30 cycles). It is believed that HTP undergoes a two‐step, three‐electron lithiation process instead of the commonly depicted two‐electron one, based on DFT calculations. In addition, HTP has much stronger intermolecular interaction in its crystal form than TTT, which is attributed to its better performance in battery. Moreover, the unit cell of HTP has channel‐like empty spaces along the [001] direction, which is favorable for a low diffusion barrier of lithium ions. In addition to the electrochemical formation of S—S bonds in batteries, the neighboring S—S bonds can be formed via photo‐oxidation of organosulfur. Shingu et al. reported the photo‐oxidation of dithiobiuret (DTB) with two neighboring tautomeric thiol/thione groups (‐SH/>C═S) that can form a disulfide bond by reversible intramolecular oxidative cyclization.^[^ ^22^ ^]^ In an acidic aqueous solution of DTB irradiated by visible light, the highest photo‐oxidation rate can reach ≈2.8 × 10^−3^ min^−1^, showing the promise of photo‐charging of a lithium battery with DTB‐like structures.

In a short summary, organosulfur molecules are feasible model compounds for understanding their electrochemical behavior in rechargeable batteries. The heteroatoms in them have profound effects on their electronic structures and battery performance. The nitrogen and phosphorous atoms can act as bonding bridges in organosulfur molecules introducing various organic groups. For example, thiuram sulfides or disulfides consist of many structures, e.g., tetramethylthiuram sulfide and di(n‐propyl)thiuram disulfide. These compounds are waiting to be explored. Coupling organosulfur cathode materials with solid or polymer electrolyte would be a viable approach to enable practical battery performance with high mass loading and long cycle life of organosulfur active materials.

### 2.2 Organosulfur Polymers

Organosulfur polymers consist of many S—S bonds with organic groups R in the structure, leading to high theoretical specific capacities.^[^ ^23^ ^]^ Usually, they have flexible and transparent properties, making them suitable for flexible batteries and devices. However, they may not have good ionic and electronic conductivities, needing to be composited with carbon materials, e.g., carbon powder, carbon nanotubes (CNTs), and graphene, etc. Recently, some new organosulfur polymers with unique structures were synthesized, which have expanded the organosulfur family and improved our understanding of these polymers in rechargeable lithium batteries.

**Figure 6:**
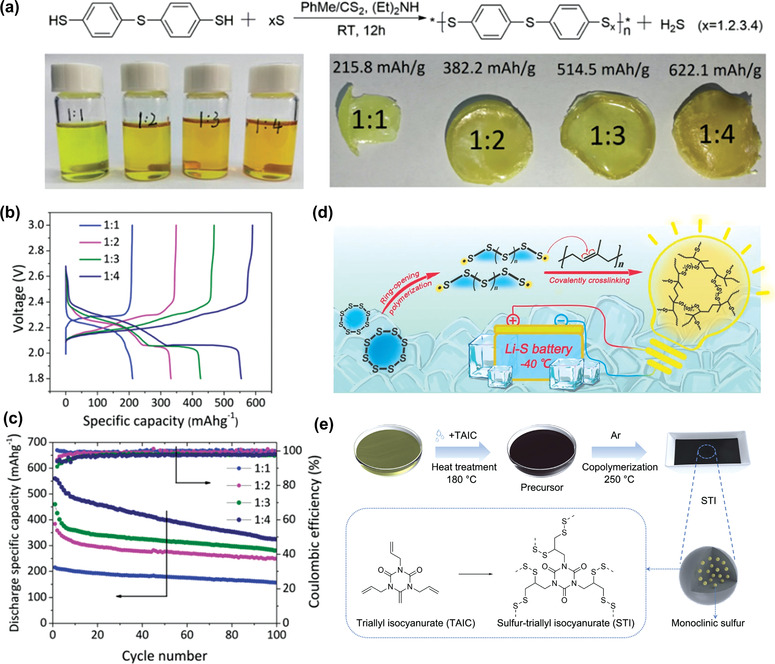
a) Synthesis of PPPS based on the condensation polymerization between 4,4′‐thiobisbenzenethiol and sulfur, the synthesized polymer solutions, and photographs of the cast polymers. b) Voltage profile of the lithium half‐cells with four polymer cathodes. c) Cycling performance of the cells at C/10 rate. Reproduced with permission.^[^ ^24^ ^]^ Copyright 2019, Royal Society of Chemistry. d) Schematic diagram of the synthesis of sulfur‐rich vulcanized clay rubber which can work at low temperature. Reproduced with permission.^[^ ^26^ ^]^ Copyright 2022, Elsevier. e) Schematic illustration of synthesis process of STI composite and its chemical structure. Reproduced with permission.^[^ ^27^ ^]^ Copyright 2020, Elsevier.

Sang et al. synthesized a new class of polyphenyl polysulfides (PPPS) by condensation reactions between 4,4′‐thiobisbenzenethiol (TBBT) and elemental sulfur with molar ratios of 1:1, 1:2, 1:3, and 1:4, as shown in **Figure** **6**a.^[^ ^24^ ^]^ The obtained materials are rubber‐like polymers with different yellow color and they are insoluble in most solvents. The PPPS with the TBBT:S ratio of 1:4 could contain sulfur chains with multiple redox active sites, leading to a high theoretical capacity of 622.1 mAh g^−1^. All these polymers show a sloping discharge voltage region followed by a flat voltage plateau at 2.1 V in lithium batteries (Figure 6b). The low voltage plateau increases with the decrease of TBBT:S ratio because increasing amount of Li_2_S is formed in the discharge. However, higher sulfur content in PPPS shows faster capacity decay in the lithium batteries (Figure 6c). The sulfur atoms bonded in the structure tend to be converted to lithium polysulfides (LiPSs) in the discharge, resulting in the dissolution and shuttle effect. Because they are insoluble in organic solvents, making the characterizations difficult. Their molecular weight and dispersity are unknown. Another series of hyperbranced polymers were reported by the same group.^[^ ^25^ ^]^ Condensation reactions between 1,3,5‐benzenetrithiol (BTT) and elemental sulfur with three different molar ratios of 1:1.5, 1:3.0, and 1:4.5 occur, leading to three polymers with hyperbranched structures (BTTP). These polymers are completely amorphous in the electrodes. In lithium batteries, they also show a sloping voltage region followed by a flat voltage part with overlapped voltage plateaus, which are due to the formation of PhS_3_Li_3_ and Li_2_S. In the cyclic voltammograms, three cathodic peaks can be seen, which are consistent with the voltage profiles. These polymers show relatively stable performance with high Coulombic efficiencies in batteries. The discharge capacities are in the range of 400–1000 mAh g^−1^, making them promising to be high‐capacity cathode materials for lithium batteries.

Recently, Wang et al. fabricated a sulfur‐rich polymer which enables all the discharged intermediates in amorphous state, promoting the battery performance at low temperature, e.g., −40 °C, as shown in Figure 6d.^[^ ^26^ ^]^ The cyclic S_8_ opens at above 159 °C, which can react with *cis*‐1,4‐polyisoprene (RUB) molecular chains to form a highly crosslinking framework. The formed sulfur‐rich vulcanized clay rubber has a high sulfur content of 75 wt%. The experimental results and DFT calculations show a new mechanism based on Li_2_S_3_ and Li_2_S_6_ intermediates. The Li cell shows a high capacity of 780 mAh g^−1^ at 1 C rate and stable cycling performance of 300 cycles at room temperature. At −40 °C, the discharge capacity is ≈67% of that at room temperature. Li et al. used the ring opening S to copolymerize with triallyl isocyanurate (TAIC), as shown in Figure 6e.^[^ ^27^ ^]^ The formed S‐triallyl isocyanurate organosulfur polymer (STI) consists of ultrafine monoclinic S particles embedded in the organosulfur polymer matrix, therefore a high S content of 90 wt% is obtained. The STI cathode shows a high initial capacity of 904 mAh g^−1^ at 0.5 C and a low capacity decay rate of 0.017% over 350 cycles at 1 C rate. Kim et al. incorporated 1D charged polypyrrole into a 2D covalent triazine framework (cPpy‐S‐CTF) synthesized in the presence of elemental sulfur.^[^ ^28^ ^]^ A high sulfur content of 83 wt% is achieved and cPpy enables a 3D nanochannel formation in the composite with high‐affinity anchoring sites toward LiPSs. The cPpy‐S‐CTF cathode shows a high discharge capacity of 1203.4 mAh g^−1^ at 0.05 C and a capacity retention of 86.8% after 500 cycles. The incorporation of charged conducting polymers is a promising strategy to enable high‐performance organosulfur polymer cathode.

As can be seen from the above studies, most organosulfur polymers are insoluble in organic solvents, making their processability difficult. Therefore, the precursors need to be injected in porous conductive substrates before they can react to form polymers. Upon cycling, the discharged and recharged products are held in the substrates leading to stable battery performance. Therefore, the substrates are critical for the battery performance of organosulfur polymers. They need to be porous to hold active materials, at the same time, they need to be very conductive.

### 2.3 Organosulfide–Metal Complexes

As electrode materials, organosulfur compounds use to have low densities, making them have low volumetric energy densities. To improve this, they need to be composited with dense materials, like metals. Another approach is to complex organosulfide with metal ions. Li et al. developed a simple method to make these complexes. Organothiols like 1,2‐benzenedithiol can react with a salt, e.g., CuSO_4_, to produce orgnaosulfide–metal complexes, like Cu‐BDT.^[^ ^29^ ^]^ When SnCl_4_ was used, Sn‐(BDT)_2_ can be synthesized. Their mass densities are about 2 mg cm^−3^, which are much higher than that (1.2 mg cm^−3^) of 1,2‐benzenedithiol. The Li/Sn‐(BDT)_2_ cell exhibits two overlapped discharge voltage plateaus and delivers a capacity of 221.2 mAh g^−1^ offering a high volumetric energy density of 973.2 Wh L^−1^. In contrast, the Li/Cu‐BDT cell exhibits four stepped discharge voltage plateaus and delivers a capacity of 234.2 mAh g^−1^ offering a high volumetric energy density of 1044.5 Wh L^−1^. Both show promising cycling stability. The charge storage occurs based on the reversible breakage/formation of sulfur–metal bonds accompanying valence changes of the metal ions. This work successfully demonstrates a plausible strategy to complex organosulfide and metal ions, which has a quite open space for further exploration.

### 2.4 Organosulfur–Carbon Composites

Organosulfur compounds usually are not electronically conductive, like elemental sulfur, which is due to the lack of conjugated structures, neither do the reduced products in batteries. To improve their conductivity, a variety of carbon materials can be composited with them, like carbon powder, CNTs, and graphene, etc. One of the successful approaches was developed by our group. We used a binder‐free CNT paper as a host/current collector for soluble organosulfur molecules. The CNT paper can be self‐weaved or purchased from commercial. It is composed of nanoscaled conductive network which is highly efficient for electron transport. In addition, they can adsorb the dissolved species including the organosulfur molecules and cycled products, making their rechargeability highly reversible. This approach is suitable for electrolyte‐soluble molecules such as dimethyl trisulfide, diphenyl trisulfide, diphenyl polysulfides, dipyridyl polysulfide, organosulfur polymers, etc.^[^ ^15^, ^24^, ^33^ ^]^ Close‐to‐theoretical specific capacity can be achieved, which allows analysis of the cycled products and revealing the redox mechanism.

**Figure 7:**
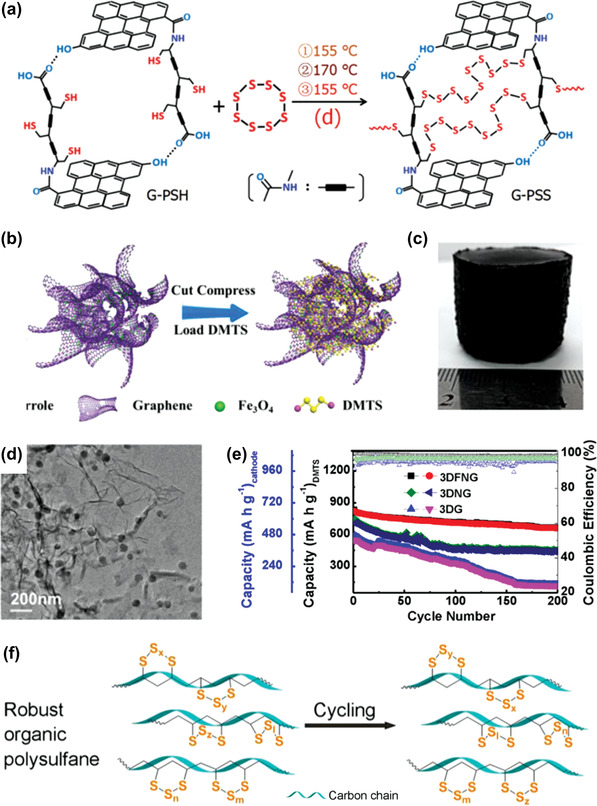
a) Schematic illustration for the synthesis of G‐PSS from G‐PSH. Reproduced with permission.^[^ ^30^ ^]^ Copyright 2019, Wiley‐VCH. b) 3DFNG composite with DMTS in it. c) Photograph of 3DFNG composite. d) TEM image of 3DFNG. e) Cycling performance of the cells with 3DG, 3DNG, and 3DFNG at C/10 rate. Reproduced with permission.^[^ ^31^ ^]^ Copyright 2019, Elsevier. f) Organic polysulfane with polycyclic sulfur grafted on the carbon before and after lithiation in battery. Reproduced with permission.^[^ ^32^ ^]^ Copyright 2019, Elsevier.

In addition to carbon matrix or additives, chemical bonding approaches have also been developed by aid of the organic groups. For example, Li et al. developed a novel cathode consisting of linear sulfur covalently attached to thiol‐rich reduced graphene oxide (rGO) as shown in **Figure** **7**a,^[^ ^30^ ^]^ in which rGO nanosheets can improve electronic conductivity and the bonding between sulfur chain and rGO reduces the formation of high‐order LiPSs and therefore the shuttle effect. By coupling with a coated separator with nitrogen and sulfur co‐doped rGO, the cell shows a high initial discharge capacity of 1364 mAh g^−1^ at 0.2 C and a reversible discharge capacity of 750 mAh g^−1^ at 1 C after 700 cycles. Recently, our group found phenyl tetrasulfide can be cycled close to 1000 times when worked with a composite electrode consisting of rGO and MoS_2_.^[^ ^34^ ^]^ The *π*–*π* stacking between the phenyl groups and rGO can significantly enhance the battery cycling performance. He et al. developed a 3D nitrogen‐doped graphene sponge decorated with Fe_3_O_4_ nanoparticles (3DFNG) as a host for dimethyl trisulfide (DMTS),^[^ ^31^ ^]^ as shown in Figure 7b–d. The nitrogen and Fe_3_O_4_ in the composite can effectively anchor DMTS and its discharged products because of their chemical interactions. Graphene and Fe_3_O_4_ provide electron transport pathways. In lithium batteries, 3DFNG is demonstrated to be a promising host for DMTS, leading to significantly improved cycling performance compared with 3DNG (3D nitrogen‐doped graphene sponge) and 3DG (3D graphene sponge) (Figure 7e). Another modification strategy has been developed by Hu et al.^[^ ^32^ ^]^ They developed a 2D organic polysulfane, an organosulfur cathode material, with a unique molecular structure of polycyclic sulfur directly substituting the carboxyls of poly(acrylic acid) and grafted on the carbon chain, as shown in Figure 7f. In a Li‐S battery, the organic polysulfane with 72 wt% sulfur content shows a high specific capacity of 891 mAh g^−1^ based on the whole composite and excellent cycling life with a capacity retention of >91% after 620 cycles at 1 C rate. Recently, Chang et al. synthesized sulfurized carbon nanotube@aminophenol‐formaldehyde with covalently linked short‐chain sulfur.^[^ ^35^ ^]^ It is in the form of mesoporous yolk‐shell organosulfur nanotubes which can effectively accommodate the volume change, expose active sites, and improve the transport of charges, leading to excellent reaction kinetics (a specific capacity of 841 mAh g^−1^ and a capacity decay of 0.06% per cycle within 500 cycles at 5 C). Hu et al. grafted organic polysulfane on porous graphene as an organosulfur electrode material.^[^ ^36^ ^]^ The mesoporous graphene provides good electrical conductivity and the organic polysulfane bonded with it shows good structural stability, leading to promising battery performance.

## 3 Organosulfur–Inorganic Hybrid Cathodes

In the process of organosulfur conversion reaction, RSLi and intermediate LiPSs are produced. To make the conversion more controllable, it is necessary to precisely locate lithiation sites during its redox process, detect specific products, and use bonding activity of the organic groups by introducing hybrid components such as inorganic nanomaterials. Through this approach, the interesting properties of organosulfur are combined with the functionalized utility of inorganic materials, leading to excellent electrochemical performance. The hybrid of organo‐inorganic covers rechargeable organosulfur, as well as a variety of inorganic nanomaterials, including metal oxides, metal sulfides, and elemental materials. The hybrid based on interatomic force also provides a facile and practical solution for promoting and boosting the electrochemical performance of batteries. In this section, we focus on hybrid cathodes composed of organosulfur and inorganic nanomaterials. In addition, the unique electrochemical properties enhanced by different interatomic forces are discussed as well.

### 3.1 Diphenyl Trisulfide‐Selenium

**Figure 8:**
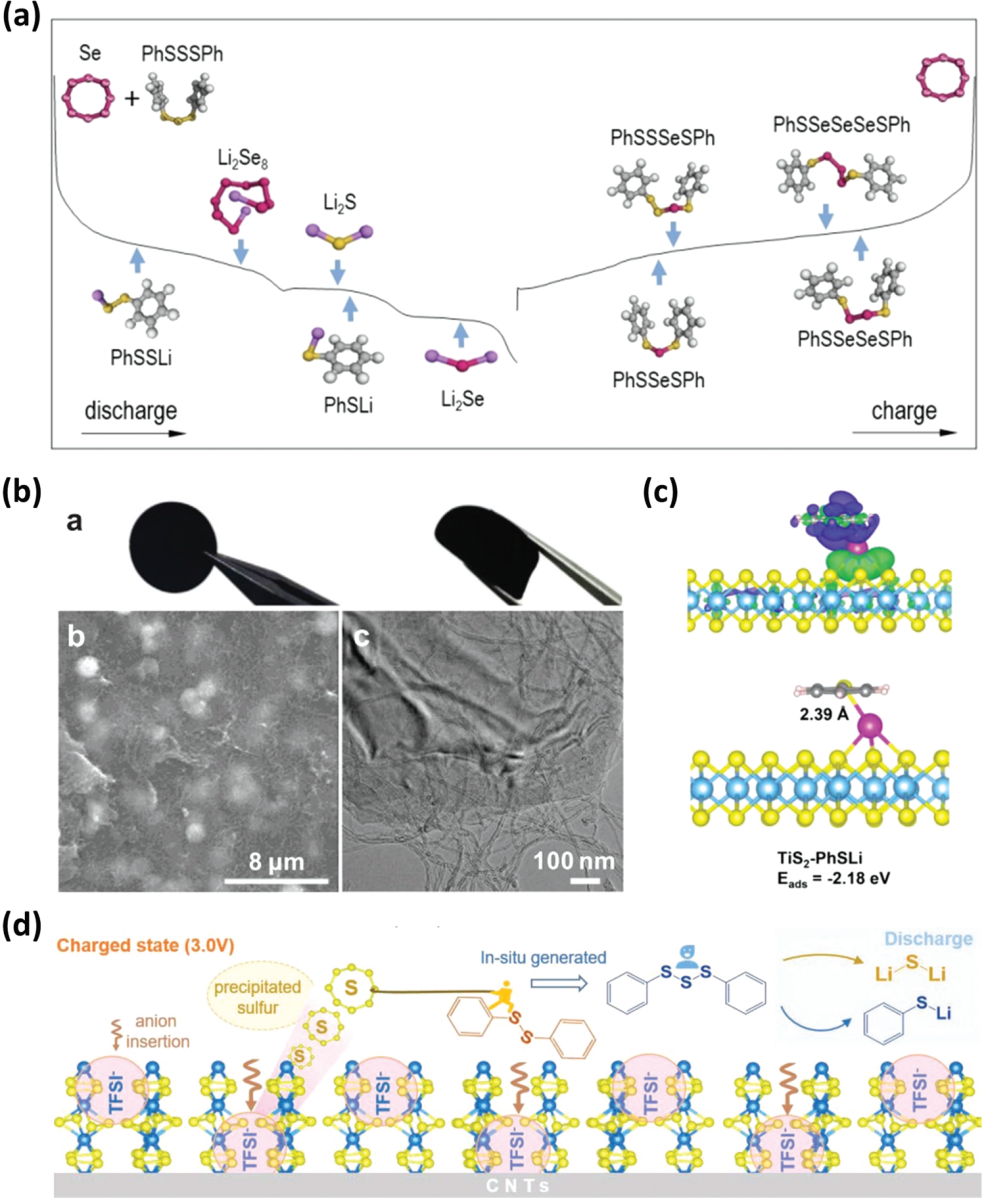
a) Redox reactions of the DPTS‐Se cathode in a Li cell. Reproduced with permission.^[^ ^37^ ^]^ Copyright 2020, Wiley‐VCH. b) Photograph, SEM image, and HR‐TEM image of the TiS_2_@CNT composite paper. c) The fully optimized adsorption configuration of PhSLi on TiS_2_ with 3D differential charge contour surface. Reproduced with permission.^[^ ^39^ ^]^ Copyright 2021, Wiley‐VCH. d) Schematic diagram of the charge–discharge mechanism of VS_4_/DPDS. Reproduced with permission.^[^ ^40^ ^]^ Copyright 2021, Wiley‐VCH.

Zhao et al. reported a hybrid cathode composed by organosulfur diphenyl trisulfide (DPTS) and inorganic selenium (Se) nanowire.^[^ ^37^ ^]^ Se nanowires were mixed with CNTs to render a self‐weaving substrate, then DPTS was introduced to form a composite electrode. The hybrid electrode shows new redox reaction mechanisms involving S—Se bonds, enhancing the specific capacity and cycling stability. The assembled organic–inorganic hybrid electrode with the advantages of conductive Se and reduction of LiPSs form several diphenyl sulfoselenide compounds containing dynamic S—Se bonds during charge process, as shown in **Figure** **8**a. Meanwhile, the spectroscopic methods, analytical techniques, and computational simulation such as DFT and Born‐Oppenheimer molecular dynamics (BOMD) supply the direct evidence to validate the formation of dynamic S—Se covalent bonds during the charge process. After the lithium products such as Li_2_Se, Li_2_S, and PhSLi are delithiated, ∙Se∙, ∙S∙, PhS∙ radicals are combined in pairs to form new products. A portion of ∙Se∙ is reconfigured into the diphenyl sulfoselenide compounds through dynamic S—Se bonds, avoiding the formation of long‐chain lithium polyselenides, which are easy to lose through the dissolution and diffusion. After the first 50 cycles, the diphenyl sulfoselenide compounds are fully formed. The Se atoms fixed in insoluble diphenyl sulfoselenide can remain electrochemically stable over prolonged cycles. The Li/DPDS‐Se battery shows a high initial discharge capacity of 471.1 mAh g^−1^ and retains a specific capacity of 325.8 mAh g^−1^ after 250 cycles. The Li/DPDS‐Se battery also shows a high Coulombic efficiency of over 99% after 250 cycles due to inhibiting the shuttle effect. In addition to organosulfide, diphenyl diselenide has also been proved to be effective to improve the Li‐Se performance by altering the redox chemistry of Se by the same research group.^[^ ^38^ ^]^ The effective combination of the organosulfide or organoselenide and Se is a promising approach to develop hybrid electrodes with superior battery performance.

### 3.2 Diphenyl Tetrasulfide‐TiS_2_

Organosulfur (R‐S*_n_*‐R, *n* ≥ 3) with long sulfur chains can enhance the discharge voltage by flexibly adjusting the functional groups, own high specific energies, and achieve low electrolyte/active material ratios. However, there are still intermediate LiPSs during the discharge process. In addition, the discharged product RSLi has the high solubility in the ether electrolyte. Hence, many organosulfur materials show unsatisfactory cycling stability and Coulombic efficiency. In regard to tackle the problems, it is vital to anchor RSLi and LiPSs and accelerate the reduction reactions within the cathode by using novel electrode configuration, such as introducing inorganic nanomaterials. 2D layered transition metal disulfides with high conductivity, large polar surface, and high catalytic activity could be good choices. TiS_2_, an ideal transporter for both lithium ion and electron, is able to facilitate reaction kinetics and adsorb LiPSs. Constructing a hybrid cathode of TiS_2_ with PTS is a effective method to promote and boost electrochemical performance of battery by Fan et al.^[^ ^39^ ^]^ The self‐woven hybrid substrate provides enough pores for physical confinement through weak binding as shown in Figure 8b, and serves as an efficient mediator for facilitating the reaction kinetics. Meanwhile, the experimental approaches such as X‐ray photoelectron spectroscopy (XPS) and DFT calculations supply the direct evidence to validate the interactions between PhSLi and TiS_2_ (Figure 8c). The hybrid PTS‐TiS_2_ cathode yields a reversible discharge capacity of 467.6 mAh g^−1^ with 99.9% capacity retention after 200 cycles at 0.5 C rate, achieving a high‐capacity retention of 81.9%. To be close to practical application, the high‐mass‐loading cells were also evaluated. The hybrid cathode with a high mass loading of 5.8 mg cm^−2^ shows a specific capacity of 444 mAh g^−1^ at 0.5 C, corresponding to the high specific energy of 901 Wh kg^−1^. The electrolyte to PTS ratio is as low as 3.8 µL mg^−1^, which can help the increase of the energy density. Through the effective combination of the organosulfur PTS and inorganic mediators TiS_2_, the hybrid cell shows the improved cycling life, and inspires us to understand the instinct interaction at molecular level, which opens a new prospect for cathode fabrication.

### 3.3 Diphenyl Disulfide‐VS_4_

The above works focus on assisting organosulfur through the introduction of inorganic nanomaterials. When focusing on the active materials of metal polysulfides, Wang et al. found more interesting phenomena assisted by organosulfur.^[^ ^40^ ^]^ Amorphous VS_4_ with specific capacity of 1196 mAh g^−1^ undergoes reduction of both cationic V and anionic S during discharge process, leading to vanadium (V) and Li_2_S. This process consists of two parts: lithium intercalation reactions at ≈1.9 V versus Li/Li^+^ and conversion reactions at below 1.0 V versus Li/Li^+^. It is found that bis(trifluoromethanesulfonyl) imide (TFSI^−^) anions can be intercalated into VS_4_ at 3.0 V in the charge process, then triggering S evolution. To avoid the escaping S changing to LiPSs, diphenyl disulfide (DPDS) was used as a sulfur receptor to capture these sulfur atoms. Hence, during the charge, DPDS changes to diphenyl trisulfide (DPTS) after S atom is evolved from VS_4_. The whole process is illustrated in Figure 8d. It can improve capacity retention and material utilization by reducing the formation/shuttle of LiPSs. A series of characterizations of sulfur evolution, such as Raman spectroscopy, especially in different charged states, XPS, provide solid evidence for this mechanism. The hybrid cathode shows a reversible capacity of 130.7 mAh g^−1^ after 300 cycles at the current density of 240 mA g^−1^.

## 4 Organosulfur Additives in Li‐S Batteries

A Li‐S battery consists of a lithium‐metal anode and an elemental sulfur cathode. During the conversion‐type reaction, Li‐S battery suffers from a number of problems, including LiPSs dissolution and migration, the instability of Li anode and SEI on it. Efforts on Li‐S battery have made great strides to conquer these issues over the years. Recently, understanding the underlying chemistry is the new target for fundamental research. By truly unlocking the potential of the Li‐S battery, the practical application of Li‐S battery will forward a solid step.

### 4.1 Dimethyl Disulfide

**Figure 9:**
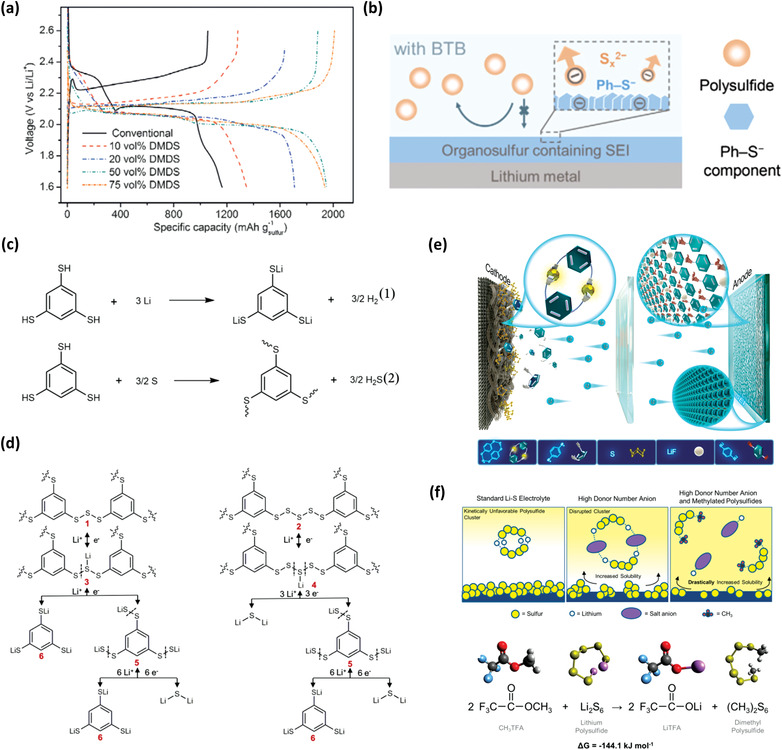
a) Initial discharge–charge voltage profiles of C/S cathodes at C/10 rate in conventional and 10–75 vol% DMDS‐containing electrolytes. Reproduced with permission.^[^ ^41^ ^]^ Copyright 2016, Wiley‐VCH. b) The schematics of the formation of SEI with BTB additive and the corresponding effect of the formed SEI in shielding LiPSs from Li anode. Reproduced with permission.^[^ ^42^ ^]^ Copyright 2020, Wiley‐VCH. c) BTT reacts with lithium metal anode and sulfur cathode. d) Schematic illustration for the Li‐S battery with 1,4‐BDT electrolyte. Reproduced with permission.^[^ ^43^ ^]^ Copyright 2021, Nature Publishing Group. e) Redox reaction processes of the Li‐S battery with BTT electrolyte. Reproduced with permission.^[^ ^44^ ^]^ Copyright 2021, American Chemical Society. f) Illustration of the theorized improvements to the Li‐S electrolyte enabled through the presence of high donor number compounds in solution, as well as high donor compounds in conjunction with organosulfur active material. Reproduced with permission.^[^ ^45^ ^]^ Copyright 2021, American Chemical Society.

The dissolution of LiPSs in liquid electrolytes and the irreversible deposition of Li_2_S on the cathode are two key issues for the rapid capacity fading of Li‐S battery. Although tremendous approaches have been made to tackle them, new approaches are still needed. Chen et al. reported a functional electrolyte system using a liquid organosulfur compound, dimethyl disulfide (DMDS), as a co‐solvent for Li‐S battery.^[^ ^41^ ^]^ Through the reversible cleavage/reformation of S—S bonds in DMDS, DMDS can afford a theoretical capacity of 570 mAh g^−1^, which helps achieve high energy density of Li‐S battery. In addition, the DMDS‐containing electrolyte enables a new electrochemical reduction pathway for sulfur cathodes, leading to improved redox kinetics and rechargeability. These effects are reflected in the performance of the battery with lower charge overpotential, as shown in **Figure** **9**a. Through a serial of electrochemical behavior analysis and characterizations, such as ^1^H NMR, scanning electron microscopy (SEM), X‐ray diffraction, and XPS, a new redox pathway is obtained, which is about the formation and subsequent reduction of soluble dimethyl polysulfide (DMPS) species. In particular, during the subsequent reduction, DMPS forms MeSLi, LiPSs, and lithium sulfide (Li_2_S), which are beneficial to the reaction kinetics and cycling performance. This work thus provides a strategy from the aspect of co‐solvent for improving the practical energy density of Li‐S batteries.

### 4.2 Organothiols

To solve the problems brought by the dissolution–precipitation redox reactions of the S cathode and the corrosion reaction on the Li metal, 3,5‐bis(trifluoromethyl) thiophenol (BTB) as an electrolyte additive is introduced into Li‐S battery by Wei et al.^[^ ^42^ ^]^ BTB provides an organosulfur‐containing SEI for shielding LiPSs and protecting Li metal anode as shown in Figure 9b, then enhancing the performance under practical conditions. Through the visualized experiments and XPS characterizations, the SEI is formed by the reaction products of BTB and Li metal, with the addition of the LiPSs. The symmetric Li–Li cell is assembled to investigate the effect of the organosulfur‐containing SEI. It shows significant enhancement on the cycling performance by shielding the Li metal and affording utilization of Li deposition. Meanwhile, Li‐S batteries with BTB achieve long lifespan and high Coulombic efficiency under practical conditions, such as high loading S cathode (4.5 mg cm^−2^), low E/S radio (5.0 µL mg^−1^), and an ultrathin Li anode (50 µm).

The interfacial instability of the Li metal anode and shuttling of soluble intermediate LiPSs in Li‐S batteries plague its applications. Most efforts have only focused on one side. Simultaneous formation of interfaces on both anode and cathode are promising solutions to fully satisfy the requirements. Guo et al. reported organothiol as a functional electrolyte additive which has thiol groups.^[^ ^43^ ^]^ The thiol groups can react with Li metal and elemental sulfur (Figure 9c), and construct functional layers. 1,3,5‐Benzenetrithiol (BTT) with the symmetrical sulfhydryl groups on the benzene ring is a typical organothiol. The in situ formed S—X (X is Li or S) originated from S—H bonds fabricate dual SEIs (D‐SEIs) on both anode and cathode, the SEI containing Li_3_‐BTT can block dendrite formation, and the possible redox reactions of the formed oligomers in the sulfur cathode are shown in Figure 9d. Through the scaling‐up reactions, the products of S—X reactions are confirmed by the characterizations of ^7^Li NMR, FTIR, Raman, XPS, and HPLC‐QTof‐MS. The reaction products protect Li anode and prevent the sulfur shuttle effect for the cathode, leading to enhanced battery performance. The Li‐S cell with BTT delivers a discharge capacity of 1239 mAh g^−1^ and high cycling stability of over 300 cycles at 1 C rate. The lithium symmetric cell with the BTT also shows the effective effect of BTT on the SEI of Li metal anode. The pouch cell supplies the results that the BTT electrolyte is beneficial for application under a low E/S ratio. Through the S—X (S or Li) bonds formation and interfacial electrochemical transformation, this work demonstrates a strategy to solve the intrinsic problems from a more chemical perspective. In addition, Lian et al. report a class of electrolyte additives, i.e., benzenedithiols (BDTs), which have three isomeric structures, including 1,2‐BDT, 1,3‐BDT, and 1,4‐BDT.^[^ ^44^ ^]^ Different conjugate diprotic structures are bridged with sulfur as different building blocks, which are analyzed by UPLC‐QTOF‐MS. After distinguishing the electrochemical performances of different concentrations for different isomer electrolytes, 0.15 m 1,4‐BDT is the best choice of the three isomers due to its high capacity for bonding sulfur and stable cycles. Figure 9e illustrates how 1,4‐BDT protects lithium metal anode and sulfur cathode. The discharge capacity of the first plateau of the cell with 1,4‐BDT is larger than that of the control Li‐S cell. It is because that the addition of 1,4‐BDT changes the route of sulfur conversion reaction and constraints the sulfur species. Hence, the Li‐S battery using the 1,4‐BDT electrolyte additive exhibits the high initial discharge capacity of 1347.1 mAh g^−1^ and stability by 909.3 mAh g^−1^ after 500 cycles at C/2 rate. The capacity fading rate is as low as 0.065% per cycle. The Li‐S pouch cell with 1,4‐BDT exhibits a capacity of 2223.9 mAh after 26 cycles. The only disadvantage of using organothiols in Li‐S batteries is that they react with Li metal and sulfur cathode releasing hydrogen and hydrogen sulfide gases, respectively. These gases can be quickly released after the electrolyte injection, which has been dealt with a gas bag in the pouch cell assembly.

Recently, Gupta et al. used methyl trifluoroacetate (CH_3_TFA) as an electrolyte additive in Li‐S batteries.^[^ ^45^ ^]^ It can react with LiPSs in situ to form lithium trifluoroacetate (LiTFA) and DMPS. The TFA anion changes solution coordination behavior, thus reducing polarization and improving discharge kinetics. At the same time, the derivatization to DMPS improves the solubility of intermediate species, enhancing the utilization of sulfur under lean‐electrolyte conditions. The theorized improvements are illustrated in Figure 9f. This strategy demonstrates a simple approach involving the formation of organosulfur molecules, thus improving the Li‐S battery performance. The functional and anionic groups can be changed, therefore there will be many possibilities which can be explored.

### 4.3 Other Organosulfur

**Figure 10:**
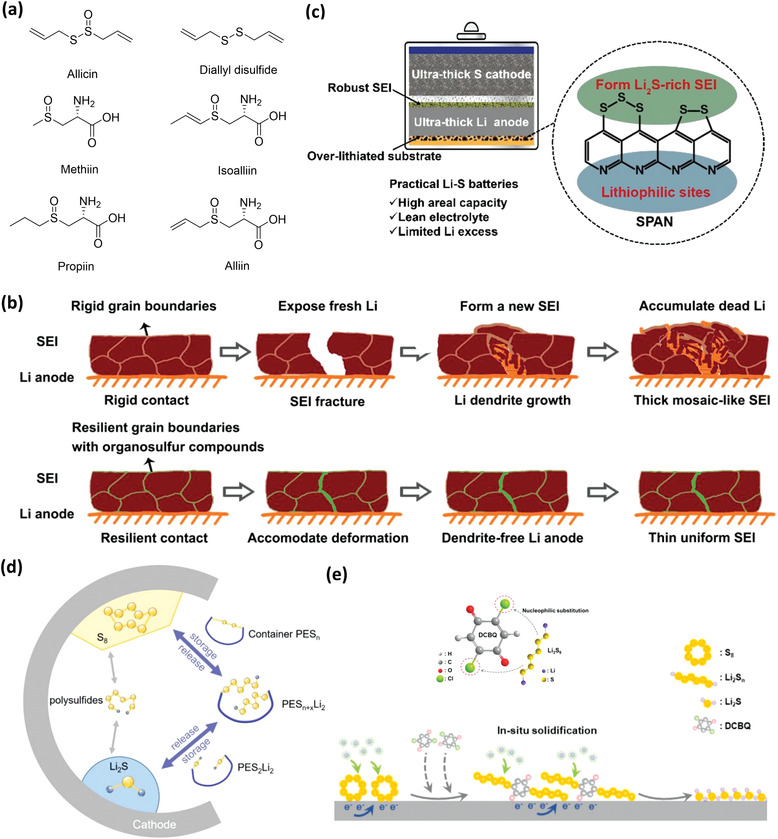
a) Organosulfur components in garlic. b) Schematics of SEI evolution and dendrite formation on the Li anode with and without organosulfur. Reproduced with permission.^[^ ^46^ ^]^ Copyright 2020, American Chemical Society. c) Schematic illustration of the configuration of a Li_2_S‐rich SEI on an ultrathick Li metal anode. Reproduced with permission.^[^ ^47^ ^]^ Copyright 2020, American Chemical Society. d) The working mechanism of sulfur container PES*_n_* in working Li‐S battery. Reproduced with permission.^[^ ^48^ ^]^ Copyright 2020, Wiley‐VCH. e) Illustration of the nucleophilic substitution reactions between polysulfides and DCBQ, and sulfur reduction process with DCBQ. Reproduced with permission.^[^ ^49^ ^]^ Copyright 2021, Elsevier.

To form stable SEI on lithium metal anode and reduce shuttle effect of LiPSs of the sulfur cathode, other organosulfur materials have also been utilized. For the Li metal anode, Boateng et al. used garlic, which consists of a variety of organosulfur compounds as shown in **Figure** **10**a, to construct a resilient SEI on the Li metal anode.^[^ ^46^ ^]^ The SEI on the bare Li anode is easy to crack and form “dead” Li upon cycling in battery, whereas SEI on the Li anode with organosulfur is stable and resilient, which can accommodate deformations leading to uniform Li plating (Figure 10b). Jiang et al. constructed a Li_2_S‐rich SEI on Li metal anode by overlithiation of sulfurized polyacrylonitrile (SPAN).^[^ ^47^ ^]^ The pyridine in SPAN acts as a lithiophilic matrix which synergistically works with the robust SEI to form an ultrathick Li metal anode, as shown in Figure 10c. When it works with a sulfur cathode with high areal capacity of 16 mAh cm^−2^, the cell shows stable cycling under a lean electrolyte of 2.2 µL mg^−1^ and low negative‐to‐positive (N/P) capacity ratio of 1.3. For the sulfur cathode, Xie et al. used a polymer organosulfur additive of di(tri)sulfide polyethylene glycol (PES*_n_*) as sulfur containers to regulate the PS intermediates in a working sulfur electrode, as shown in Figure 10d.^[^ ^48^ ^]^ It can reversibly store sulfur species by forming organosulfur during charge and release them during discharge, improving reaction kinetics and cycling performance. Chen et al. developed an in situ solidification strategy by using 2,5‐dichloro‐1,4‐benzoquinone (DCBQ) as an electrolyte additive. It can covalently bond with LiPSs via nucleophilic substitution reactions forming a solid S‐DCBQ organosulfur polymer which can effectively impede the polysulfide dissolution and migration, as shown in Figure 10e. It also can accelerate the lithium ion transport and reaction kinetics, improving the battery performance.

## 5 Organosulfur Materials in Other Metal Batteries

Orgnosulfur materials have been extensively studied in rechargeable lithium batteries. Their intriguing redox mechanisms and the effects of different functional groups R on their electrochemical behaviors have been revealed. Based on the previous understanding, interests in their applications in other battery systems have grown obviously. Interesting phenomena have been observed and they have shown unique properties in improving battery performance. Below we list a few examples, which can serve as the beginning of application of organosulfur materials in the wide battery systems.

### 5.1 Redox Flow Batteries

**Figure 11:**
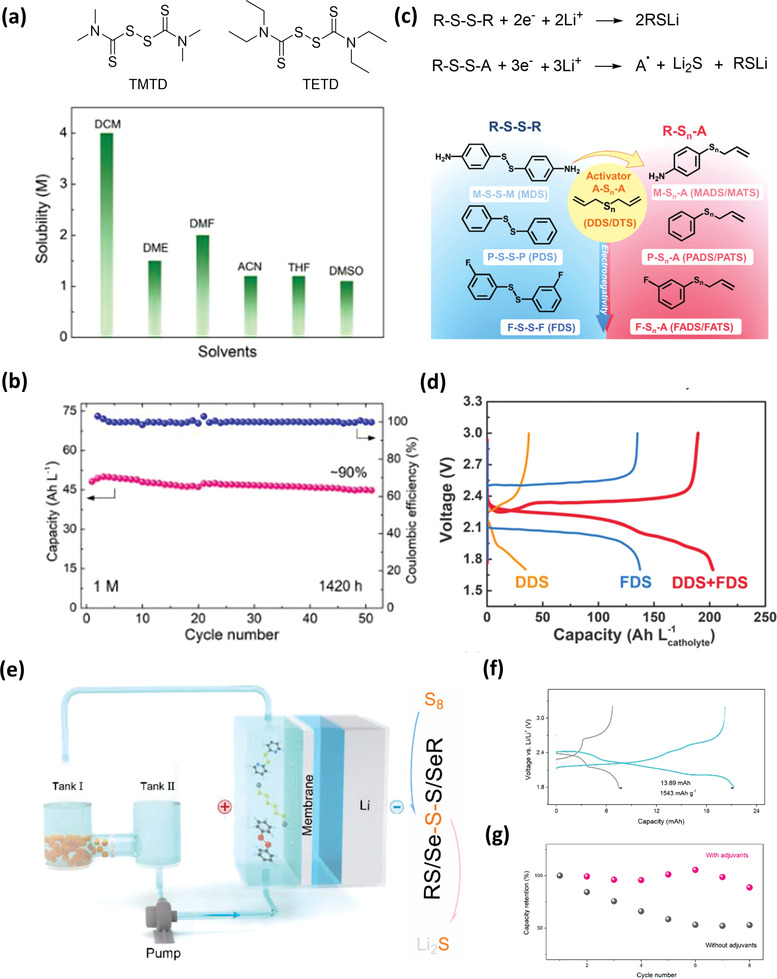
a) Molecular structures of TMTD and TETD, and the solubility of TETD in various organic solvents. b) Cycling performance of 1.0 m TETD at 0.3 mA cm^−2^. Reproduced with permission.^[^ ^51^ ^]^ Copyright 2021, Wiley‐VCH. c) The schematic of asymmetric organosulfides synthesis and its redox mechanism. d) Voltage profiles of DDS, FDS, and DDS+FDS at 0.1 mA cm^−2^. Reproduced with permission.^[^ ^52^ ^]^ Copyright 2019, Elsevier. e) Schematic illustration of a Li‐S RFB with DpyDS and PhSeSePh as adjuvants. f) Voltage profiles of the 15 × 10^−3^ m DpyDS+PhSeSePh before/after adding 9.0 mg sulfur in tank at 0.3 mA cm^−2^. g) Comparison of capacity retention rate of the Li‐S RFB with/without adjuvants at 0.6 mA cm^−2^. Reproduced with permission.^[^ ^53^ ^]^ Copyright 2021, Royal Society of Chemistry.

Organosulfur materials, especially small molecules such as DMTS, DPDS, DPTS, and DpyDS are highly soluble in organic solvent, which makes them suitable as active materials or adjuvants in catholyte for nonaqueous redox flow batteries (RFBs). Tetramethylthiuram disulfide (TMTD) with relatively low molecular weight was first adopted as the active material of a nonaqueous Li‐organosulfur RFB by Wang et al.^[^ ^50^ ^]^ However, perhaps due to the shuttle of active materials or the irreversible chemical reaction between organosulfur lithium compound (discharged product) and carbonate electrolyte, the Li‐organosulfur RFB shows rapid decay of capacity. Recently, Zhang et al. use tetraethylthiuram disulfide (TETD) as an active material for nonaqueous RFBs.^[^ ^51^ ^]^ Comparing with TMTD, TETD has high solubility (>1 m, **Figure** **11**a) in polar organic solvents, e.g., acetonitrile (ACN) and 1,2‐dimethoxyethane (DME). In addition, a cell with solid ceramic separator is adopted to assess the intrinsic cycling stability of TETD, which can avoid the shuttling of active materials between electrodes. The TETD electrolyte with the concentration of 1.0 m renders a high reversible capacity of 50 Ah L^−1^, and after 50 cycles, the capacity retention is 90% (Figure 11b). To improve the achievable capacity and reaction kinetics of organodisulfides, Weng et al. reported a new strategy based on asymmetric allyl‐activation.^[^ ^52^ ^]^ One organic functional group (R) in the symmetric organodisulfides (R‐S‐S‐R) is replaced with an allyl (A) to form asymmetric organosulfides (R‐S*_n_*‐A). The R‐S*_n_*‐A can be simply prepared in the supporting electrolyte by mixing phenyl disulfides (MDS, PDS, and FDS) with allyl‐based organosulfur (DDS and DTS), as shown in Figure 11c. In general, one R‐S‐S‐R can take up 2 electrons. For R‐S‐S‐A, the A—S bond will be cleaved in the discharge process arising from the facile generation of allyl radials, therefore, one R‐S‐S‐A can take 3 e^−^ (Figure 11c). Furthermore, asymmetric organosulfides show faster kinetics than symmetric organodisulfides, a cell of FDS+DDS with lower overpotential than FDS and DDS is obtained, as shown in Figure 11d.

Besides as active materials for nonaqueous RFBs, organosulfur also can serve as adjuvants in Li‐S RFBs to improve the sulfur material utilization. Chen et al. used DpyDS and diphenyl diselenide (PhSeSePh) as adjuvants into a Li‐S RFB (Figure 11e).^[^ ^53^ ^]^ Some sulfur atoms can be inserted into the organodisulfide/diselenide structures to generate new active materials (RSSSR/RSSSeR) during the discharge–charge process. The inserted sulfur atoms can be transformed into Li_2_S directly in the absence of long LiPSs (e.g., Li_2_S_6_ and Li_2_S_8_), which accelerate redox kinetics of sulfur and improve the sulfur material utilization. In addition, this strategy can effectively remove the passivation of Li metal anode via self‐healing process due to the presence of DpyDS and PhSeSePh in the circulating catholyte. Because of the above reasons, a superior sulfur utilization of 92% (1543 mAh g^−1^ based sulfur mass) is achieved in a continuous Li‐S RFB (Figure 11f). After eight cycles, the capacity retention of the cell with DpyDS and PhSeSePh is 88.8%, which is much higher than 53% capacity retention of a single Li‐S RFB without DpyDS and PhSeSePh, as shown in Figure 11g.

### 5.2 Deep Eutectic Electrolyte in Li/LiFePO_4_ Battery

**Figure 12:**
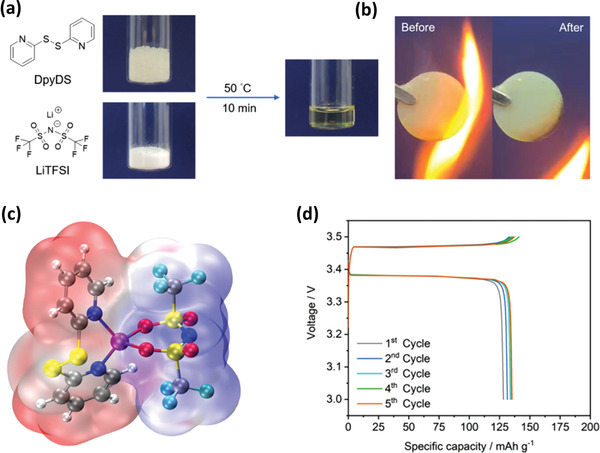
a) Preparation of DEEs with DpyDS and LiTFSI. b) Flammability test of the DEE in a glass fiber membrane. c) Molecular electrostatic potential energy surface of the DEE. (d) Voltage profiles of a Li/LiFePO_4_ cell with DEE as electrolyte at C/10 rate. Reproduced with permission.^[^ ^54^ ^]^ Copyright 2021, Wiley‐VCH.

As active materials in lithium batteries, organic functional groups in organosulfur have profound effects on their electrochemical performance in the above works. Interestingly, Song et al. found that the Li^…^N interaction between DpyDS and LiTFSI is similar with Li^…^O interaction, which can cause the deep eutectic effect.^[^ ^54^ ^]^ The deep eutectic electrolytes (DEEs) can be simply prepared by mixing DpyDS with LiTFSI at different molar ratios (**Figure** **12**a), in which the pyridine moieties of DpyDS act as Lewis base and the Li ions of LiTFSI serve as Lewis acid. This class of DEEs is inflammable, as shown in Figure 12b. The existence of Li^…^N interaction and formation mechanism are confirmed through Raman spectra and DFT calculations (Figure 12c). At 50 °C, the DEE with the DpyDS:LiTFSI ratio of 4:1 (DEE‐4:1) exhibits an electrochemical stability window between 2.1 and 4.0 V versus Li/Li^+^ with the ionic conductivity of 1.5 × 10^−4^ S cm^−1^. A Li/LiFePO_4_ cell with DEE‐4:1 as the electrolyte shows stable voltage profiles (Figure 12d).

### 5.3 Sodium Metal Batteries

**Figure 13:**
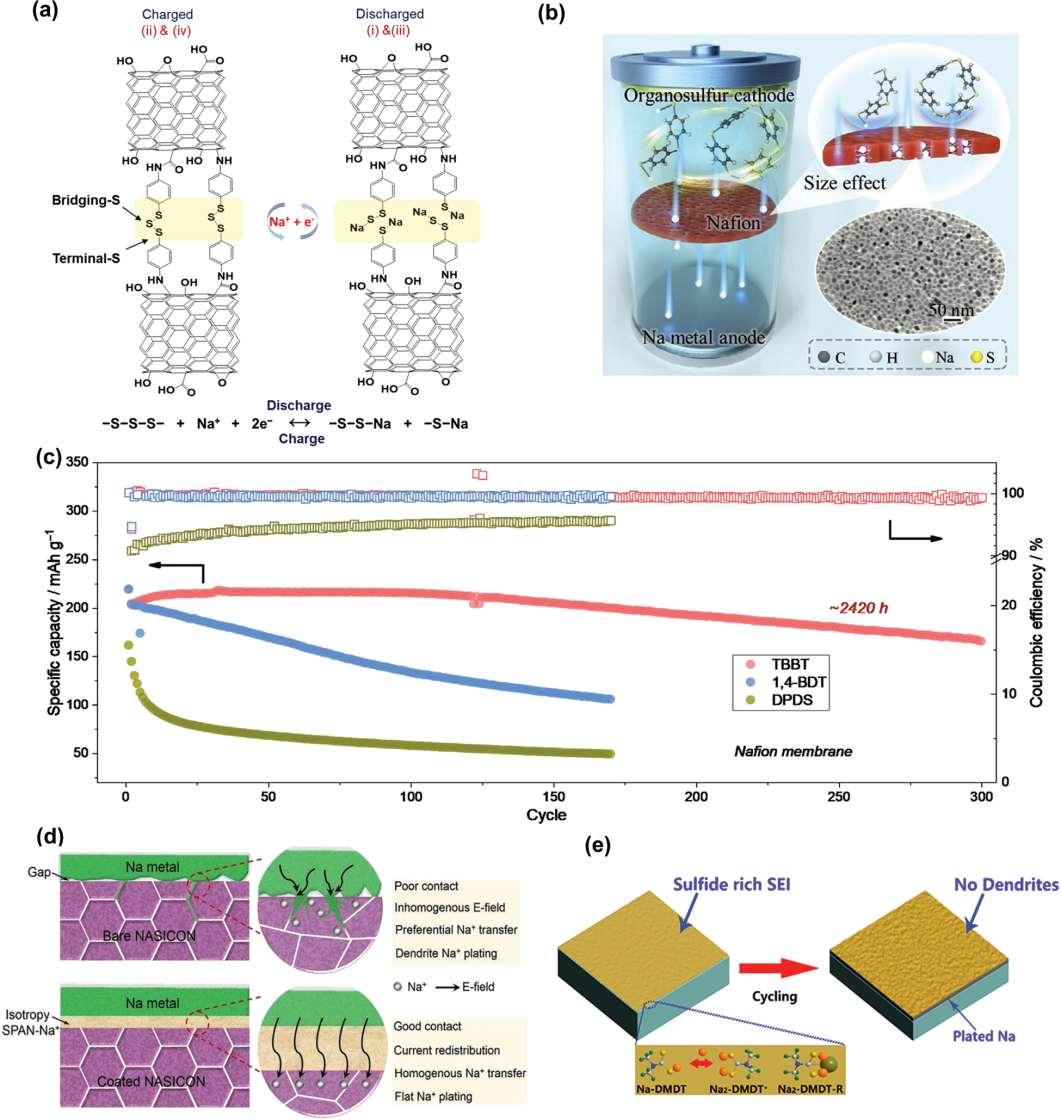
a) Proposed redox mechanism of SOS−OCNT. Reproduced with permission.^[^ ^55^ ^]^ Copyright 2021, American Chemical Society. b) The cell configuration with the size effect of organosulfur. c) Cycling performance of Na/organosulfur cells with the Nafion membranes. Reproduced with permission.^[^ ^56^ ^]^ Copyright 2021, Wiley‐VCH. d) Schematic illustrations of the Na deposition behavior of with and without SPAN layer. Reproduced with permission.^[^ ^57^ ^]^ Copyright 2021, Wiley‐VCH. e) Schematic illustrations of the evaluation of the SMA surface morphology in the TMTD‐based electrolyte. Reproduced with permission.^[^ ^58^ ^]^ Copyright 2021, American Chemical Society.

Room temperature sodium‐sulfur (Na‐S) battery is one of the promising choices for the next generation of low‐cost and large‐scale energy storage systems. However, severe shuttle effect of sodium polysulfides results in low utilization of sulfur and rapid capacity decay. Organosulfur compounds can work as active material for sodium storage by reversible cleavage and formation of S—S bonds either intramolecularly or intermolecularly in sodium batteries. Jana et al. reported surface redox‐active SOS‐OCNT synthesized through the formation of −CO−NH−R and −C−NH−R chemical bonds between the oxygen functional groups of organosulfur‐modified ozone‐treated CNTs (OCNT) and SOS (4‐aminophenyl trisulfide). The formed organosulfur moiety (−S−S−S−) between CNTs serve as active sites for sodium storage, as shown in **Figure** **13**a.^[^ ^55^ ^]^ It is found that the organosulfur moiety undergoes a two‐electron transfer process instead of four, which is confirmed by in situ and ex situ analysis. In another word, the discharge state is the low‐order sodium polysulfide (−S−S−Na/−S−Na) and the −S−S−S− bond can be formed reversibly during^[^ ^57^ ^]^ recharge. The Na‐organosulfur cathode exhibits the high energy density of 27 Wh kg^−1^ and long cycle life for 50 000 cycles.

Tang et al. researched the electrochemical behavior of three organosulfur cathodes in sodium batteries and found that the size of organosulfur molecules has profound effect on their electrochemical behavior and battery performance (Figure 13b).^[^ ^56^ ^]^ Three organosulfur compounds including 4,4′‐thiobisbenzenethiol (TBBT), 1,4‐benzenedithiol (1,4‐BDT), and DPDS were selected. In the sodium cells with Celgard separators, severe shuttle effect is observed. In contrast, the ion selective membrane (Nafion) can effectively block the shuttle of TBBT and 1,4‐BDT, but not DPDS. In the charge process, TBBT and 1,4‐BDT are converted to large cyclic oligomers which can be effectively blocked by the nanoscaled ion channels in Nafion membrane, leading to good cycling performance. The van der Waals sizes of the corresponding oligomers of TBBT and 1,4‐BDT are 13.16 and 11.46 Å, which are larger than that (11.18 Å) of DPDS. The Na cell with TBBT shows a high capacity retention of 77% over 300 cycles (2420 h), as shown in Figure 13c.

Furthermore, organosulfur can also be used as electrolyte in sodium batteries. In order to improve the wettability and reduce the interfacial resistance of Na_3_Zr_2_Si_2_PO_12_ ceramics, Miao et al. reported a green and simple powder polishing method to modify the pyrolytic PAN (pPAN) intermediate layer on the surface of Na superionic conductor (NASICON) ceramics. The introduced sulfur can trigger the dehydrogenation and cyclization of PAN, thus rendering SPAN layer on the surface of NASICON particles (Figure 13d). Isotropic and delocalized groups allow the SPAN interlayer to uniformly redistribute the electrons/ions transport and maintain excellent cycling stability. A novel cell configuration is designed including the SPAN cathode and the SPAN‐based electrolyte to improve cathode/SSE interfacial compatibility. The Na/SPAN‐NASICON/SPAN Na‐organosulfur full cell shows a reversible discharge capacity of 357.1 mAh g^−1^ for over 100 cycles.

Additionally, organosulfur also can be used as electrolyte additives in Na batteries. Zhu et al. reported the tetramethylthiuram disulfide (TMTD) as the carbonate‐based electrolyte additives,^[^ ^58^ ^]^ which can form the organic sulfur salt layer to protect sodium anode interface during cycling as shown in Figure 13e. Therefore, the sulfide‐rich SEI can inhibit the formation of sodium dendrite and promote the uniform deposition of Na ions. The Na symmetric cell with the TMTD‐added electrolyte shows sable cycling performance of 1600 h at 0.25 mAh cm^−2^. When paired with Prussian Blue cathode, the cell delivers a reversible capacity of 86.2 mAh g^−1^ with a capacity retention of 80% after 600 cycles at 4 C rate.

### 5.4 Magnesium Metal Batteries

Magnesium (Mg) metal anode has several benefits: 1) dendrite growth on Mg metal anode has little or no risk; 2) abundant Mg resources and low cost; 3) the high specific (2205 mAh g^−1^) and volumetric (3833 mAh cm^−3^) capacity and the low reduction potential. However, traditional magnesium‐sulfur (Mg‐S) batteries suffer from poor cycling stability and reversibility. Very few studies were carried out on the application of organosulfur materials in Mg batteries. Kaland et al. reported the dipentamethylene thiuram tetrasulfide (PMTT) as the cathode,^[^ ^59^ ^]^ which exhibits a high discharge capacity of 295 mAh g^−1^ in Mg battery. During the reduction process of PMTT, the S—S bonds are preferentially attacked and broken, then the structure of Mg pentamethylene dithiocarbamate (Mg‐(PMDTC)_2_) is formed. In addition, the PMTT‐derived sulfur/mesoporous carbon composite also was prepared that displays excellent rate performance (185 mAh g^−1^ at 500 mA g^−1^) and 76% capacity retention after 100 cycles.

## 6 Summary and Outlook

**Figure 14:**
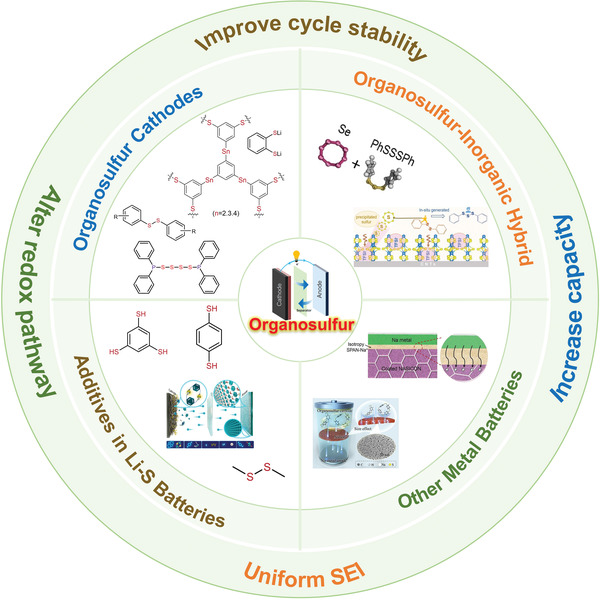
Summary illustration of the organosulfur materials in rechargeable metal batteries.

In summary, organosulfur materials can be regarded as promising functional components in the next‐generation rechargeable batteries owing to their abundant resources, environmental benignity, tunable structures, and low cost. Accordingly, in this review we discuss in detail the structure–property–performance relationship of organosulfur recently investigated, as summarized in **Figure** **14**. First, we provide recently studied organosulfur cathode materials in rechargeable Li batteries, including molecules and polymers. The organosulfide–metal complexes and organosulfur–carbon composites are briefly introduced, which endow organosulfur with unique electrochemical properties and enhanced battery performance. The organosulfur–inorganic hybrid materials are presented, organosulfur can play unique roles in the hybrids and can participate in electrochemical redox processes of the inorganic components leading to the enhancement of discharge capacity and cycling stability. Furthermore, recent researches on the organosulfur as electrolyte additives are also summarized. Organosulfur can participate in the formation of SEI, thereby protecting the lithium metal anode and inhibiting the formation of lithium dendrites. Meanwhile, in Li‐S batteries, organosulfur can be combined with LiPS to alleviate the shuttle effect. Finally, organosulfur can also be used in other types of batteries including redox flow batteries, Na and Mg batteries. In Na batteries, it has also been confirmed that the type of separator and the size of organosulfur molecules are the factors that affect the cycling performance. Additionally, the DpyDS and LiTFSI can form DEEs, which can be used as electrolytes for rechargeable lithium batteries.

Current studies have shown the great potential of organosulfur in battery applications, there are still lots of opportunities and challenges. The followings list some perspectives on the future development of organosulfur: 1) design and synthesize novel organosulfur molecules with defined structures, e.g., novel organosulfur molecules with more active sites for high specific capacity. In addition, new synthetic methods are needed to afford large‐scale synthesis; 2) apply advanced characterization techniques to clarify the redox mechanism of organosulfur. Up to now, the redox intermediates of organosulfur are still difficult to capture. Therefore, in order to better understand their reaction mechanism, more advanced characterization techniques such as in situ spectroscopies are needed; 3) investigate organosulfur in various battery systems, such as zinc, aluminum, and calcium batteries. They have clear advantages of low cost and high capacity/energy density. To date, only limited efforts have been focused on other metal batteries; 4) develop solid‐state batteries with organosulfur materials. Some organosulfur molecules can be adapted in solid‐state batteries to improve interfacial properties. For example, DEEs could be used as conductive glues in solid‐state battery electrodes. In addition, the discharged products of organosulfur can conduct ions, which can be used as salt additives in solid‐state electrolytes; 5) hybrid organosulfur with other inorganic and organic materials. It has been confirmed that organosulfur can be involved in the redox process of metal polysulfides. However, only few inorganic materials have been studied in the hybrids. Other metal polysulfides like MoS*_x_* or even organic electrode materials should be coupled with organosulfur. New redox mechanisms may be discovered. In addition to the above perspectives, the electrochemical conversions of organosulfur materials in batteries can be utilized to synthesize new compounds like BDPPTS. Joint efforts between chemists and electrochemists are needed to discover more organosulfur materials with diverse potential applications.

Organosulfur possesses many advantages and the family of organosulfur is large. Their potential has been shown in this review and more endeavors need to be made in this area. It is believed more interesting results and intriguing mechanisms will be discovered. There is still some way to go before organosulfur materials can be used in commercial batteries. During the application process, two outstanding issues need to be considered. For organosulfur molecules as cathode materials, solid or polymer electrolytes are needed, which can block their crossover and reduce the risk. In addition, since organosulfur materials are not lithiated, therefore Li metal anodes are needed which add additional challenges for the practical application of organosulfur. In order to replace the unsafe lithium metal anode, it is also necessary to consider the development of suitable electrolytes to enable organosulfur be paired with lithiated graphite anode. For other battery systems, such as Li‐S batteries, the commercial use of organosulfur is also promising. Maybe organothiols used as electrolyte additives could become the first trials in commercial batteries. In addition, the introduction of Se—S bonds could bring unexpected benefits. Although some challenges need to be overcome, we believe organosulfur will play a critical and diverse role in the future battery systems and beyond.

## Conflict of Interest

The authors declare no conflict of interest.
